# Retraction: Effects of environmental impact labels on the sustainability of food purchases: Two randomised controlled trials in an experimental online supermarket

**DOI:** 10.1371/journal.pone.0309440

**Published:** 2024-08-22

**Authors:** Christina Potter, Rachel Pechey, Michael Clark, Kerstin Frie, Paul A. Bateman, Brian Cook, Cristina Stewart, Carmen Piernas, John Lynch, Mike Rayner, Joseph Poore, Susan A. Jebb, the PLOS ONE Editors

After this article [1] was published, the authors noted an error regarding the validity of the results presented in Study 2.

Specifically, incorrect nutrition labels were displayed in the experimental supermarket for Study 2 in error.

In light of this error, which impacts the reliability and validity of the results presented in Study 2, the authors retract the article. The error had no impact on impact Study 1, and an updated version of the article reporting Study 1 alone has been published [2].

All authors agreed with the retraction.
